# Management Challenges of Severe, Complex Dyskinesia. Data from a Large Cohort of Patients Treated with Levodopa-Carbidopa Intestinal Gel for Advanced Parkinson’s Disease

**DOI:** 10.3390/brainsci11070826

**Published:** 2021-06-22

**Authors:** József Attila Szász, Viorelia Adelina Constantin, Károly Orbán-Kis, Ligia Ariana Bancu, Marius Ciorba, István Mihály, Előd Ernő Nagy, Róbert Máté Szász, Krisztina Kelemen, Mihaela Adriana Simu, Szabolcs Szatmári

**Affiliations:** 1 2nd Clinic of Neurology, Târgu Mureș County Emergency Clinical Hospital, 540136 Târgu Mureș, Romania; szaszneuro@yahoo.com (J.A.S.); constantin.viorelia@umft.ro (V.A.C.); istvan.mihaly@umfst.ro (I.M.); krisztina.kelemen@umfst.ro (K.K.); szabolcs.szatmari@umfst.ro (S.S.); 2 “George Emil Palade” University of Medicine, Pharmacy, Science and Technology of Târgu Mureș, 540142 Târgu Mureș, Romania; ligiabancu@yahoo.com (L.A.B.); ciorba_im@yahoo.com (M.C.); elod.nagy@umfst.ro (E.E.N.); szaszrobert48@gmail.com (R.M.S.); 3 Doctoral School, ”Victor Babes” University of Medicine and Pharmacy Timisoara, 300041 Timișoara, Romania; 4 1st Clinic of Internal Medicine, Târgu Mures County Emergency Clinical Hospital, 540142 Târgu Mureș, Romania; 5 Department of Gastroenterology, Târgu Mures County Emergency Clinical Hospital, 540142 Târgu Mureș, Romania; 6 Laboratory of Medical Analysis, Clinical County Hospital Mures, 540142 Târgu Mureș, Romania; 7 Department of Neurology II, “Victor Babes” University of Medicine and Pharmacy Timisoara, 300041 Timișoara, Romania; simu.mihaela@umft.ro; 8 ”Pius Branzeu” Emergency Clinical County Hospital, 300723 Timișoara, Romania

**Keywords:** advanced Parkinson’s disease, levodopa-carbidopa intestinal gel, diphasic dyskinesia, motor complications

## Abstract

Background: In the advanced stages of Parkinson’s disease (APD), complex forms of dyskinesia may severely impair the patient’s quality of life. Objective: In the present study, we aimed to analyze the evolution under LCIG therapy of the most important motor fluctuations and complex disabling dyskinesias, including diphasic dyskinesia. Methods: In this retrospective study, we analyzed the characteristics of patients with APD who had at least 30 min of diphasic dyskinesia (DID) in 3 consecutive days, were considered responders and were treated with LCIG in our clinic. Patients were evaluated before and after PEG and at 6, 12 and 18 months, when the changes in the therapy were recorded, and they completed a 7-point Global Patient Impression of Improvement (PGI-I) scale. Results: Forty patients fulfilled the inclusion criteria—out of which, 34 performed all visits. There was a substantial difference between the calculated and real LCIG (1232 ± 337 mg vs. 1823 ± 728 mg). The motor fluctuations and most dyskinesias improved significantly after starting LCIG, but an increasing number of patients needed longer daily administrations of LCIG (24 instead of 16 h). Conclusions: Patients with APD with complex dyskinesias must be tested in dedicated hospitals, and they need a special therapeutic approach. The properly adapted LCIG treatment regarding the dose and time of administration completed with well-selected add-on medication should offer improvement for patients who want to or can only choose this DAT vs. others.

## 1. Introduction

Parkinson’s disease (PD) is the second-most frequent, progressively worsening neurodegenerative disorder. Among the drugs approved for the symptomatic treatment of PD, levodopa (LD) remains the gold standard [1]. As the disease progresses, the efficacy of traditional oral/transdermal medication is gradually decreased and becomes unpredictable. Motor complications (fluctuations and dyskinesias) resulting from uneven drug absorption, narrowed therapeutic window and drug level fluctuations severely impair the patient’s autonomy, social interactions and quality of life (QoL) [2,3]. The continuous delivery into the upper intestine of the levodopa-carbidopa intestinal gel (LCIG) provides a more stable plasma level compared to oral LD administration in patients with nonoptimal control of motor fluctuations. Therefore, this delivery method is considered to be a long-term treatment option for motor fluctuations in patients with advanced PD (APD) who are refractory to oral medication [4,5,6,7]. Evidence derived from randomized and open-label clinical trials has proven that LCIG infusion is also effective in the treatment of levodopa-induced dyskinesias (LID), despite an overall increase in the mean daily levodopa dose. This benefit is thought to be related to a change in LD pharmacokinetics with continuous drug delivery (LCIG therapy has a substantial anti-dyskinetic effect and could be an alternative also for APD patients with dyskinesia as a major symptom/cause of disability) [8,9,10]. We do not have knowledge of studies that have evaluated the long-term management of patients with severe motor fluctuations and complex disabling dyskinesias, including diphasic dyskinesia (DID).

## 2. Materials and Methods

We conducted a retrospective evaluation of all patients with APD treated at the 2nd Neurology Clinic in Târgu Mureș with LCIG between 1 June 2011 and 31 May 2017. Our main purpose was to identify patients with severe motor fluctuations and complex, disabling dyskinesia (peak dose, diphasic dyskinesia and end of dose dystonia), as well long term follow-up after LCIG therapy initiation. During the 6-year period covered by this analysis, we enrolled all APD patients still responding to LD who (i) reported at least 2 h/day of off periods (with ≥2 off episodes/day, except early morning akinesia) with or without dyskinesias; (ii) who were on ≥3 stage on the Hoehn and Yahr scale during on periods and (iii) who received LD at least four times daily in some combination with dopamine agonists (DA), monoamine oxidase B inhibitors (MAO-Bi), catechol-O-methyl transferase inhibitors (COMTi) and/or amantadine. Partial data on the selection and (in-hospital) evaluation process, the spectrum of motor complications and the characteristics of the last dopaminergic treatment have been previously published [11,12,13,14].

A two-step evaluation was performed for each patient. During the first visit, we organized a personalized training for them (when appropriate, also together with relatives), with video demonstrations from the clinic’s video library to facilitate the recognition and interpretation of each phase of motor complications. Previously, patients were informed that this visit will be longer in duration, in order to capture the transition from phase on to off and vice versa. Additionally, PD medication was administered according to a previously established schedule, and the effects were recorded (with written consent) for a better understanding of the various motor complications and the temporal correlation with the medication administration schedule. At the second evaluation visit (baseline) patients presented with journals completed in the last 3 days before the visit. The inclusion criteria for this special group of patients with complex dyskinesias were: at least 30 min of diphasic dyskinesia (DID) on 3 consecutive days, considered responders and treated with LCIG. Testing for the efficacy of LCIG therapy was performed under continuous hospitalization. The calculation of the estimated doses of LCIG (calculated/theoretical LCIG) was done according to the recommendations in the literature [15]. Patients were evaluated at discharge and at 6, 12 and 18 months, respectively (±one month), and, of course, whenever needed. At each visit, the changes in therapy were recorded (add-on medication, changes in the dose of LCIG and the daily duration of administration). In order to facilitate the evaluation of patients and the quantification of dyskinesias, especially DID, on the occasion of the first visit, we trained the patients and/or their relatives regarding the recognition of their different phases. Thus, to differentiate peak dose dyskinesia from diphasic dyskinesia, we used the concept of early incomplete on with dyskinesia and late incomplete on with dyskinesia (we considered this concept necessary given that, after initiating continuous treatment with LCIG, the recognition of dyskinesias according to the oral administration of LD was no longer possible).

At discharge, and at each visit, patients also completed a 7-point Global Patient Impression of Improvement (PGI-I) scale in order to rate their perceived total improvement of PD and whether or not this improvement was due entirely to the drug treatment: (1) very much improved, (2) much improved, (3) minimally improved, (4) no change, (5) minimally worse, (6) much worse and (7) very much worse.

Patient data was analyzed using either the chi-square test or nonparametric one-way ANOVA (Kruskal–Wallis), followed by Dunn’s multiple comparison test (when appropriate), unless otherwise specified. Values are presented as the mean ± SD, unless otherwise specified.

## 3. Results

Of the 311 patients with motor fluctuations initially examined, only 286 patients (110 with dyskinesias) were present at the second evaluation (inclusion visit). Of these, 125 cases were considered suitable for device-aided therapies (DAT) [11,12,13,14]—out of which, 83 (66.4%) also presented dyskinesias, and 43 (34.4%) were enrolled in the group with complex, disabling dyskinesia (peak dose, diphasic dyskinesia and end of dose dystonia) associated with severe motor fluctuations. After testing the efficiency of LCIG with a naso-jejunal probe, three patients were considered non-responders, and in the end, PEG-J was performed on 40 patients. An overview of the profiles of these patients is presented in Table 1.

**Figure 1:**
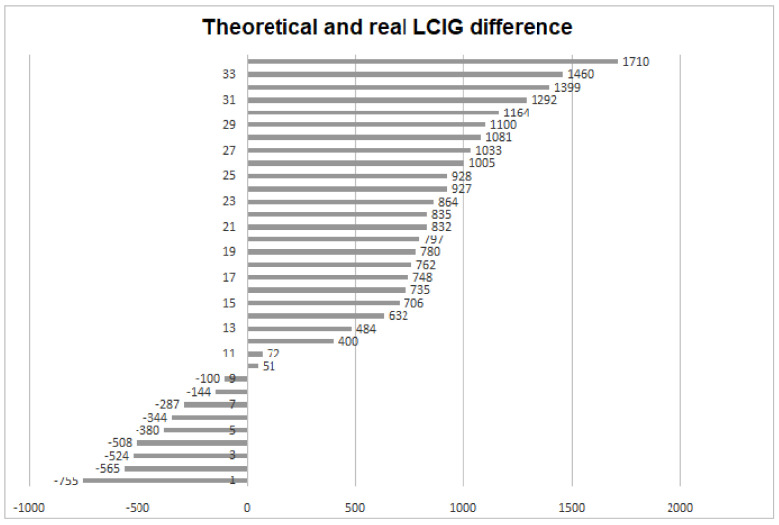
A considerable difference was found between the calculated/theoretical and real-life LCIG doses.

Given the complex clinical picture, the titration period for significant improvement was longer and more difficult, and there was a substantial difference between the calculated/theoretical LCIG (1232 ± 337.6 mg) and that with which the patients were discharged (real LCIG, 1823 ± 728.4 mg) (Figure 1).

**Figure 2:**
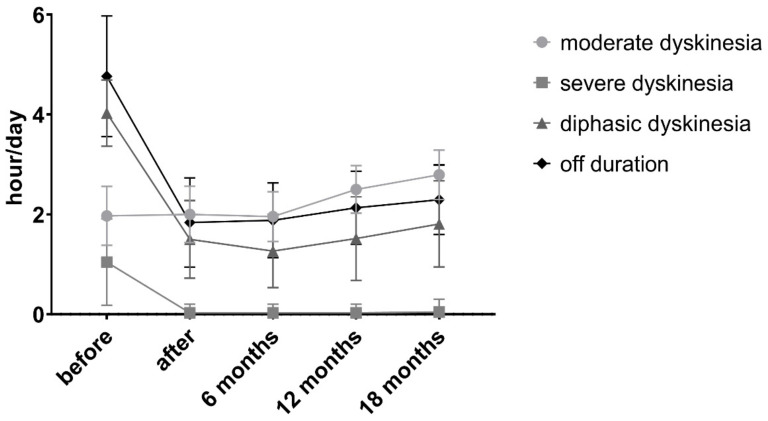
Evolution of the motor complications before and after PEG. Statistical evaluation of the changes is detailed in Table 2.

At the end of the 18-month period, only 34 patients remained in the study. The profiles of these patients, information regarding the LCIG doses, add-on medication, motor complications and clinical picture are summarized in Table 2 and Figure 2. The reasons why six patients stopped the treatment are as follows: one death due to sudden cardiac arrest, one patient developed pulmonary thromboembolism, one patient had frequent falls that led to a femoral fracture, and eventually, the family members decided to discontinue treatment, one patient presented psychotic episodes and repeatedly sectioned the tubes, one patient became noncompliant without proper family support and one patient was placed in a nursing home without the possibility for further reliable management of the LCIG treatment.

**Figure 3:**
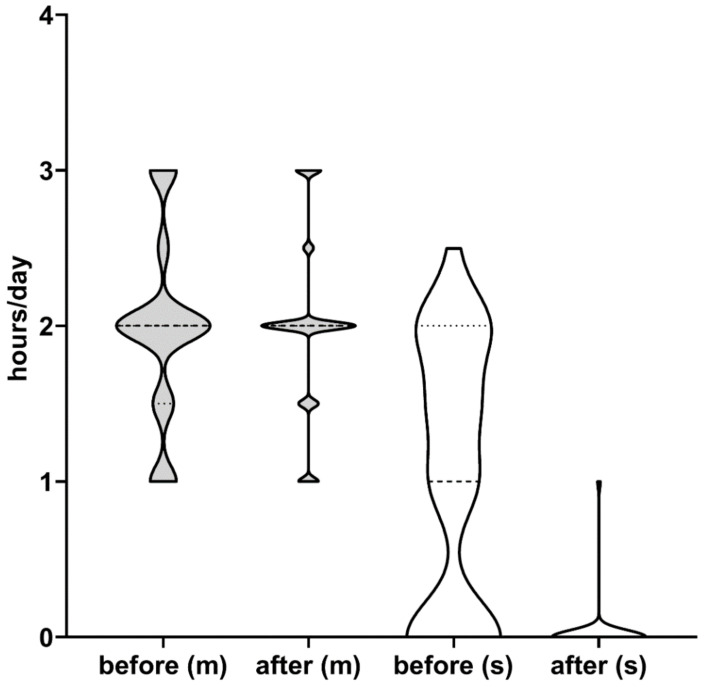
Violin plot of moderate (grey, marked with m) and severe (white, marked with s) dyskinesia before and after PEG. Dashed line—median and dotted line—quartiles. Note the drastic reduction of severe dyskinesia after PEG, an effect that was sustained for 18 months.

After the PEG, a slight increase of moderate dyskinesia (h/day) was coupled with a near-lack of severe forms (Figure 3). Off hours/day, as well as diphasic dyskinesia, dystonia and early morning akinesia, were also significantly reduced (Figure 2 and Table 2).

## 4. Discussion

In the present survey, we present the experience of an Eastern-European Center with expertise in movement disorders with a high patient turnover, where the only DAT available for APD is LCIG. According to national regulations, the initiation of therapy is done only in a hospital—more specifically, in university clinics, where there are multidisciplinary teams with dedicated training [7,16,17]. Deep brain stimulation (DBS) can be used only in a small number of cases, and apomorphine for continuous infusion has not yet been registered.

Despite the growing number of therapies with proven symptomatic efficacy, substitution therapy with LD (the gold standard in PD therapy) is still key for the best clinical improvements at all stages of the disease. Long-term LD administration, disease progression and pulsatile drug delivery are considered important risk factors for the development of motor and nonmotor complications that significantly impair the patients QoL [18,19,20,21]. After 4 to 5 years of treatment, at least 40% of patients develop significant motor fluctuations and dyskinesias, and the rate of motor complications exceeds 90% after a 10-year disease duration [22]. These disadvantages should be alleviated by an association to LD drugs of different add-on therapies that improve the bioavailability (in variable associations and dosages), such as the third-generation catechol-O-methyltransferase inhibitor (COMTi) opicapone, the selective and reversible inhibitor of monoamino oxidase B (MAOB) and glutamate release modulator safinamide [23,24,25,26]. Gastrointestinal motility, often impaired in APD (mainly gastroparesis, which is a major destabilizing factor of LD plasma levels) [27], can be circumvented by administering LD with an inhaler (in the form of an aerosol). This delivery method’s ability to quickly alleviate off periods was proven both in clinical trials and everyday practice [28]. It cannot be confirmed at this time whether the magnitude of the clinical benefits of these preparations (used mainly in patients with mid- to late-stage fluctuating PD) is fully reproduced in APD. However, their values may be increasingly appreciated by the patients who will not accept DAT.

Several add-on treatments, such as safinamide and opicapone, are not available in Romania, nor is tolcapone, extended release amantadine, LD inhalation powder or subcutaneous apomorphine injection for the rapid relief of off episodes [17,29].

Under these circumstances, as a logical consequence of the significantly limited therapeutical options available for clinicians to treat APD, there is a possibility that some patients may start available device-aided therapies in an earlier disease stage than recommended in the worldwide current clinical practice.

The continuous delivery of LCIG directly into the proximal jejunum via percutaneous endoscopic gastro-jejunostomy (PEG-J) provides reliable absorption and more stable plasma concentrations of LD [30,31]. Compared to the conventional oral levodopa therapy, LCIG has demonstrated a significant reduction of off time, increase of on time without troublesome dyskinesias and has also been shown to improve the nonmotor complaints commonly associated with chronic oral LD therapy [5,6,32]. The tolerability profile of LCIG is generally comparable with that of oral therapies, with the exception of events related to the delivery system and its placement [33]. Furthermore, a recent systematic review and meta-analysis demonstrated that LCIG has comparable effects to STN-DBS on motor functions for APD, with acceptable tolerability [34].

The standard duration of infusion of LCIG for patients with APD treatment is considered to be around 16 h [4], but it can be administered up to 24 h if medically justified, in patients with specific therapeutic needs (e.g., nocturnal akinesia) [35]. In our patients, right from the beginning of the treatment, during the dose adjustment in the hospital, we had to use continuous administration (24/24 h) in 13 out of 34 cases in order to obtain an optimal control of the symptoms. During the follow-up period in three other patients, we switched to continuous administration, and overall, there was a tendency to increase the number of LCIG administration hours (of the 11 initial patients with 16-h/day administration, only seven were on this treatment regimen after 18 months from initiation). Additionally, another clinical observations suggested that a 24-h LCIG infusion may further improve the symptoms (LD-unresponsive freezing of gait, poorly controlled nocturnal fluctuations or early morning off symptoms and uncontrolled troublesome dyskinesias may also benefit from a 24-h infusion) [36,37,38].

Dyskinesias have been traditionally classified according to their motor phenomenology, as well as their relationship with the LD dosage (supposed plasma concentrations). The most common form of LID is ”peak-dose” dyskinesia or inter-dose dyskinesia, which consists of a combination of choreiform and dystonic movements and occurs when LD reaches the peak plasma concentration (parallel with the maximal clinical benefit). Chorea that may be generalized or affect the more affected side or upper body (truncal and upper-limb—predominant chorea) may occur during the whole therapeutic window of the LD dose cycle [39]. More complex response patterns may also occur when the dopamine plasma concentration levels rise (becoming effective) or decline after each dose and typically consist of repetitive, stereotypic movements of the legs (lower-body—predominant chorea or DID) or abrupt fluctuations between a good antiparkinsonian response (with or without LID) and a severe parkinsonian motor state (“unpredictable on–off fluctuations” or “sudden off”). Conversely to peak-dose dyskinesias, diphasic dyskinesias (also referred to as beginning- and end-of-dose or transitional dyskinesia) tends to have a more variable topographical distribution and to be reported as more disabling compared with peak-dose dyskinesia [39] (see, also, Supplemental Video S1). This phenomenon is considered to be a marker indicating that the patient is not fully on [40]. Diphasic dyskinesias are frequently accompanied by repetitive alternating movements, pain, dystonia and restlessness. Some patients are susceptible to both dyskinetic patterns but experience each at different times. Additionally, in connection with LID, we have to mention off-period dystonia, characterized by fixed and painful postures more frequently affecting the feet but which can be segmental or generalized in distribution (See Supplemental Video S2). A combination of any of these three types or all of them may be observed in some patients throughout the entire LD cycle (see, also, Supplemental Video S3) [39]. The factors associated with a higher incidence and/or early development of LID include the LD dosage, disease severity and duration, a young age at PD onset and female gender [22]. It is not possible at present to explain the clinical observation according to which a proportion of PD patients never develop dyskinesias during their lifetime exposure to LD [39].

On the other hand, LID can be considered a marker of the effectiveness of LD replacement therapy [40]. However, we must accept that the disability given by bradykinesia is, in fact, replaced by the disability given by LID. It is generally accepted that mild/moderate dyskinesias are more easily accepted by patients than periods of severe off (a tendency to increase LD doses can be frequently observed, even if this implies an increase in the duration/severity of LID) [2]. In a recent publication in which we analyzed the time required to accept DAT, we found that those with severe dyskinesia with prolonged duration are willing to switch faster to DAT [41]. Conversely, PD patients who have never exhibited dyskinesia may be undertreated, with a potentially greater global disability, because the onset and offset of therapeutic and dyskinetic thresholds have been demonstrated to be similar. APD patients may thus alternate between severe bradykinesia, rest tremor, gait disorders, freezing or other parkinsonian features and LID (peak-dose and/or diphasic) without a “clean”, fully functional on state in between [39].

Randomized clinical trials have confirmed that LCIG reduced the off time and increased the on time without increasing troublesome dyskinesias and improved the QoL [4,5]. The anti-dyskinetic effect of LCIG was mainly reported in post hoc analyses. Thus, a post hoc analysis of the GLORIA registry highlighted that the severity of dyskinesia, and its associated pain improved significantly in all patients but, to a greater extent, in those with an increased duration of dyskinesia at baseline (≥4 h/day). The periods of off and the score of motor symptoms (UPDRS, Part III) were significantly reduced compared to the initial moment, regardless of the previous degree of dyskinesia. The LCIG treatment significantly improved the quality of life regardless of the duration of dyskinesia at the baseline [42]. A 6-month open-label pilot study, showing a 47% reduction of on time with troublesome dyskinesia, proved that LCIG has a substantial anti-dyskinetic effect and could also be an alternative, long-term treatment option for APD patients with dyskinesias. Moreover, LCIG leads to significant improvements in nonmotor symptoms, in daily living activities and QoL in patients with motor complications [8].

There are a few publications about the effect on dyskinesias of the 24-h LCIG therapy; in one such study, daytime dyskinesia was reduced in 75% (9/12) of patients following treatment with 24-h therapy [37]. Nevertheless, some patients treated with LCIG may develop disabling diphasic dyskinesia in the morning and at the end of the day, when L-dopa levels rise and wear off, respectively [43,44,45]. Therefore, the onset of drug-related dyskinesias with mixed characteristics in APD patients under continuous administration of LD can be presumed.

Under these circumstances (especially for clinicians whose only DAT option is LCIG), several dilemmas arise:

1. Does the expected benefit of improving motor fluctuations compensate for the potential worsening of dyskinesias?
2. In patients with pre-existing troublesome dyskinesias (especially if they have DID), as well as those with low compliance and/or high expectations, how long should one try to follow the 16-h LCIG regimen before starting, with all the necessary precautions, the 24-h administration?
3. How do we deal with add-on medication, especially amantadine (discontinuation, maintenance and starting the drug if it was not used previously)?
4. Do DIDs that occur during treatment with LCIG have the same pattern as those that occurred during treatment with oral LD?
5. How do we interpret the change of the LID profile observed in clinical practice during the LCIG treatment, and does this change the imposed additional measures?
6. What is the importance of individual susceptibility, and does it impose additional measures?
7. In cases of patients that are eligible for both LCIG and DBS (patients with APD under 70 years, with moderate or severe motor fluctuations and dyskinesias or without depression or dementia), if a patient opted for LCIG, considering the treatment as “less invasive” compared to DBS, what is the degree and type of dyskinesia that should suggest (if available) a switch to DBS?

The profiles of the 40 patients with APD that were assessed in the present retrospective analysis included complex, disabling motor complications: average off periods of 4.80 ± 1.19 h/day associated, with a moderate peak dose of dyskinesias of 1.98 ± 0.71 h/day and biphasic dyskinesias of 4.05 ± 0.69 h/day. The latter value is the sum of the periods of early incomplete on and late incomplete on (concepts introduced to define those periods in which the patient is not in fully on, and the switch to LCIG therapy deprives the patient of the recognition of dyskinesias, depending on the doses of oral LD). Additionally, clinical practice has shown that the classification of dyskinesias based on the “classical” pattern described in the literature [39]—namely, predominance in the upper limbs and trunk in the case of peak dose dyskinesia and lower limbs in diphasic dyskinesia—is not always possible.

Additionally, 22 patients had severe peak dose dyskinesias before the PEG (1.61 ± 0.46 h/day) and 12 had end-of-dose dystonia (1.83 ± 0.94 h/day). A freezing phenomenon was present in 20 patients. At the evaluation before discharge (at the end of the testing phase and fine-tuning after PEG-J), we noticed a decrease of the off periods to 1.84 ± 0.9 h/day, which was associated with a reduction of both severe dyskinesias (out of 22 cases, only one patient was left with 1 h/day) and DID (1.5 ± 0.78 h/day). In parallel, we noticed a slight increase in the duration of moderate dyskinesias, which denoted a change in the profile of dyskinesias, a phenomenon observed throughout the 18 months. To our knowledge, this phenomenon has not been described in the literature. Overall, we consider the improvement as resulting from the reduction of off periods and the increase of on periods without troublesome dyskinesias (even if we have to acknowledge a change in the profile of dyskinesias, as described above) to be consistent and long-lasting. Additionally, we must emphasize the near-complete remission of freezing in more than half of the patients (out of the 20 patients with freezing, only nine still presented with freezing after LCIG, and at 6 and 12 months, only seven patients remained with this motor complication).

Regarding the time of initiation, the LCIG titration (the length of the naso-jejunal testing phase) and the management of the previous add-on dopaminergic medication were largely left at the clinician’s personal option and experience. There is a lack of clear recommendations from experts, although these would be particularly important for increasing the long-term safety and efficacy and lowering the discontinuation rate. It is rational to assume that polypharmacy and complex treatment regimens may contribute to nonadherence. The recently published COSMOS study (a large, multinational, retrospective, cross-sectional, observational study, the first dedicated to investigating a comedication use with LCIG and the potential usability of LCIG as a monotherapy) provided evidence that LCIG monotherapy is a feasible and effective long-term treatment option for symptoms of APD. Additionally, patients with preserved combination therapy also experienced considerable reductions in comedication use [46]. The decreasing trend of combination therapy could also be seen in this special category of our patients with APD, with the exception of amantadine, the utilization rate of which steadily increased from 18% to 56% during the 18-month follow-up period.

Although there is robust evidence for the clinical benefits of LCIG in patients with APD, there are no studies assessing the safety and efficacy of high doses (≥2000 mg), and information on the use of high-dose LCIG is limited. In a recently published report, the rate of adverse events was higher in patients requiring ≥ 2000-mg/day LD compared with patients who received <2000 mg/day but was still consistent with the established safety and tolerability profile of LCIG [47]. Overall, the continuous administration of LCIG is beneficial to APD patients who require very high doses of levodopa. In our study group, the dose of LCIG at discharge (real) was 1823 ± 728.4 mg (significantly higher than the calculated/theoretical dose of 1232 ± 337.6 mg), and 17 patients received ≥2000 mg/day. The incidence of adverse events in this category was comparable to the previously reported data and was similar to those described in the literature [17], but we want to emphasize that four of the six patients who discontinued treatment (including the patient with repeated psychotic episodes) received LD doses of over 2000 mg/day. There were no other notable psychiatric complications during the evaluated period.

This retrospective analysis targeted a particular population of patients with APD that are less-analyzed in dedicated clinical trials. The new available therapeutic possibilities open new opportunities in the management of cases with motor complications, even those with complex dyskinesias. However, a series of new challenges appeared, given the tendency toward profile changes despite the continuous dopaminergic stimulation. We consider our results representative. In previous publications, we documented that the therapeutic strategies used in the treatment of PD in our region are similar to those found in the literature [48,49]. We want to reaffirm that, in Romania, the suitability of DAT can only be assessed in a university teaching hospital setting. We also want to highlight the fact that, in Romania, a number of well-established add-on therapies that are well-suited to the treatment of APD are still not available. Additionally, in our region, the only available DAT is LCIG. Therefore, this therapeutic solution is also often considered for eligible patients for whom DBS may have been the first treatment option. Therefore, we believe that all these factors, when considered together, faithfully reflect not only the Romanian but, maybe, the Central-Eastern European situation of challenges, limitations and difficulties but, also, therapeutic success and robust expertise in the long-term management of APD with complex motor complications. The retrospective nature of this study, incomplete or missing data and the lack of knowledge regarding the involvement of the caregiver, as well as the impossibility to determine the plasma LD level, can be considered limitations of this work.

## 5. Conclusions

Patients with APD with complex dyskinesias need a special therapeutic approach, ideally tested in hospitals. A properly adapted LCIG treatment regarding the dose and time of administration completed with well-selected add-on medication should offer improvements for patients who want to or can only choose this DAT. Targeted, clinical trials are needed to assess the changes observed in the dyskinesia profile and to identify possible additional measures that could further improve the quality of life of these patients and could increase the efficiency and safety of LCIG.

## Figures and Tables

**Table 1:** Profiles of the patients before PEG.

	DID (*n* = 40)	
Gender (*n*, %)		
Male	12, 30%	
Female	28, 70%	
Age (years, mean ± SD)		*p* = 0.92
All patients	64.13 ± 7.21	
Male	63.25 ± 8.79	
Female	64.50 ± 6.56	
Disease duration until LCIG infusion		
Years, mean ± SD	12.63 ± 5.01	
Years, median	11.50	
MMSE (mean ± SD)	25.83 ± 1.62	
Hoehn and Yahr score (Mean ± SD)		<0.0001
on state	3.25 ± 0.44	
off state	4.5 ± 0.50	
Off duration (hours, mean ± SD)	4.80 ± 1.19	
Peak dose dyskinesia (hours, mean ± SD)		
Mild/moderate	1.98 ± 0.71	
Severe	1.56 ± 0.58	
Diphasic dyskinesia (hours, mean ± SD)		
Early deficient on	2.84 ± 0.55	
Late deficient on	1.21 ± 0.32	
Total	4.05 ± 0.69	
Dystonia (hours, mean ± SD)	1.83 ± 0.88	
Early morning akinesia (hours, mean ± SD)	0.98 ± 0.35	
Delayed on (*n*, %)	35, 87.5%	
No on (*n*, %)	16, 40%	
Sudden off (*n*, %)	20, 50%	
Freezing (*n*, %)	23, 57.5%	
Levodopa until LCIG infusion		
Frequency (x/day, mean ± SD)	5.38 ± 1.23	
Dose (mg/day, mean ± SD)	756.3 ± 273.0	
DA (*n*,%)	34, 85%	
Pramipexole (*n*; mean ± SD mg)	19; 2.23 ± 0.76	
Ropinirole (*n*; mean ± SD mg)	16; 14 ± 4.9	
Rotigotine (*n*; mean ± SD mg)	10; 7.6 ± 2.1	
COMT-I (*n*,%)	26, 65%	
Amantadine (*n*,%)	16, 40%	
MAO-I (*n*,%)	33, 82.5%	
LCIG calculated	1232 ± 337.6	
LCIG real	1823 ± 728.4	

**Table 2:** Profiles of the patients after PEG.

*n* = 34	Before PEG	After PEG	6 Months	12 Months	18 Months	*p*
LD (LCIG) dose (mean ± SD)	--	1763 ± 671	1700 ± 607	1712 ± 603	1720 ± 595	ns
LD (LCIG) infusion	--					
administration hours		19.64 ± 3.57	19.65 ± 3.57	19.88 ± 3.58	20.24 ± 3.47	0.8105
Mean ± SD		18	18	18	18	
Median		11	11	10	7	
16 h (*n*)		10	10	10	12	
18 h (*n*)		13	13	14	15	
24 h (*n*)						
Hoehn and Yahr score (Mean ± SD)						
on state	3.26 ± 0.45	3	3	3	3.03 ± 0.17	<0.0001 ^1^
off state	4.5 ± 0.51	3.82 ± 0.46	3.82 ± 0.46	3.82 ± 0.46	3.91 ± 0.51	<0.0001 ^2^
Off duration	4.76 ± 1.21	1.84 ± 0.89	1.88 ± 0.75	2.13 ± 0.73	2.29 ± 0.7	<0.0001 ^3^
(*n*, hours, mean ± SD)						
Peak dose dyskinesia (hours, mean ± SD)						
Mild/moderate (*n* = 34)	1.97 ± 0.6	2 ± 0.56	1.96 ± 0.5	2.5 ± 0.48	2.79 ± 0.49	<0.0001 ^4^
Severe (*n* = 22)	1.61 ± 0.46	0.03 ± 0.17	0.03 ± 0.17	0.03 ± 0.17	0.04 ± 0.26	<0.0001 ^5^
Diphasic dyskinesia (hours, mean ± SD)	4.03 ± 0.66	1.5 ± 0.78	1.27 ± 0.73	1.51 ± 0.84	1.81 ± 0.86	<0.0001 ^6^
Dystonia	12, 1.83 ± 0.94	9, 1.06 ± 0.39	9, 0.94 ± 0.46	9, 1.22 ± 0.44	9, 1.22 ± 0.36	0.0310 ^7^
(hours, mean ± SD)						
Early morning akinesia (hours, mean ± SD)	31, 1.06 ± 0.21	0, 0	6, 0.58 ± 0.2	11, 0.59 ± 0.20^4^	12, 0.88 ± 0.23	<0.0001 ^8^
Freezing (*n*)	20	9	7	7	8	0.0018 ^#^
DA (*n*,%)	See Table 1.	10, 29%	11, 32%	11, 32%	11, 32%	0.9916 ^#^
Amantadine (*n*,%)	See Table 1.	6, 18%	12, 35%	18, 53%	19, 56%	0.0041 ^#^
MAO-I (*n*,%)	See Table 1.	13, 38%	12, 35%	15, 44%	15, 44%	0.8620 ^#^
PGI-I (mean ± SD)	--	1.61 ± 0.55	1.71 ± 0.58	1.71 ± 0.58	2.29 ± 1.00	0.0040 ^9^
Very much improved (*n*)		14	12	12	7	
Much improved (*n*)		19	20	20	14	
Minimally improved (*n*)		1	2	2	11	
No change (*n*)		0		0	0	
Minimally worse (*n*)		0		0	2	
Much worse (*n*)		0		0	0	
Very much worse (*n*)		0		0	0	

^#^ Chi-square test. ^1^ Before PEG significantly different (<0.0001) when compared to either after PEG or 6 months, 12 months and 18 months. No other groups show a paired significance. ^2^ Before PEG significantly different (<0.0001) when compared to either after PEG or 6 months, 12 months and 18 months. No other groups show a paired significance. ^3^ Before PEG significantly different (<0.0001) when compared to either after PEG or 6 months, 12 months and 18 months. No other groups show a paired significance. ^4^ Before PEG vs. 12 months (0.0017), before PEG vs. 18 months (<0.0001), after PEG vs. 12 months (0.0029), after PEG vs. 18 months (<0.0001), 6 months vs. 12 months (0.0010) and 6 months vs. 18 months (<0.0001). All other pairs were not significant. ^5^ Before PEG significantly different (<0.0001) when compared to either after PEG or 6 months, 12 months and 18 months. No other groups show a paired significance. ^6^ Reduction per patient number was not significant (chi-square, *p* = 0.1468), but ANOVA was significant when comparing hours/day. Paired comparisons were significant between before PEG and after PEG or 6 months, 12 months and 18 months (<0.0001). ^7^ Significant between before PEG and 6 months (0.0278). ^8^ Significant between before PEG vs. after PEG (<0.0001), as well as before PEG vs. 6 months (0.0009) and before PEG vs. 12 months (<0.0001). ^9^ Significant when after PEG was compared to 18 months (0.0057), between 6 months and 18 months (0.0359) and between 12 and 18 months (0.0359).

## Data Availability

The data presented in this study are available on request from the corresponding author. The data are not publicly available due to privacy reasons.

## Supplementary Materials

The following are available online at https://www.mdpi.com/article/10.3390/brainsci11070826/s1, Supplemental Video S1: Patient RI. Supplemental Video S2: Patient VE. Supplemental Video S3: Patient OM.

## References

[1] Ferreira J.J., Katzenschlager R., Bloem B.R., Bonuccelli U., Burn D., Deuschl G., Dietrichs E., Fabbrini G., Friedman A., Kanovsky P. (2013). Summary of the recommendations of the EFNS/MDS-ES review on therapeutic management of Parkinson’s disease. Eur. J. Neurol..

[2] Pirtošek Z., Bajenaru O., Kovács N., Milanov I., Relja M., Skorvanek M. (2020). Update on the Management of Parkinson’s Disease for General Neurologists. Parkinson’s Dis..

[3] Odin P., Ray Chaudhuri K., Slevin J.T., Volkmann J., Dietrichs E., Martinez-Martin P., Krauss J.K., Henriksen T., Katzenschlager R., Antonini A. (2015). Collective physician perspectives on non-oral medication approaches for the management of clinically relevant unresolved issues in Parkinson’s disease: Consensus from an international survey and discussion program. Park. Relat. Disord..

[4] Olanow C.W., Kieburtz K., Odin P., Espay A.J., Standaert D.G., Fernandez H.H., Vanagunas A., Othman A.A., Widnell K.L., Robieson W.Z. (2014). Continuous intrajejunal infusion of levodopa-carbidopa intestinal gel for patients with advanced Parkinson’s disease: A randomised, controlled, double-blind, double-dummy study. Lancet Neurol..

[5] Antonini A., Poewe W., Chaudhuri K.R., Jech R., Pickut B., Pirtošek Z., Szasz J., Valldeoriola F., Winkler C., Bergmann L. (2017). Levodopa-carbidopa intestinal gel in advanced Parkinson’s: Final results of the GLORIA registry. Park. Relat. Disord..

[6] Juhász A., Aschermann Z., Ács P., Janszky J., Kovács M., Makkos A., Harmat M., Tényi D., Karádi K., Komoly S. (2017). Levodopa/carbidopa intestinal gel can improve both motor and non-motor experiences of daily living in Parkinson’s disease: An open-label study. Parkinsonism Relat. Disord..

[7] Băjenaru O., Ene A., Popescu B.O., Szász J.A., Sabău M., Mureşan D.F., Perju-Dumbrava L., Popescu C.D., Constantinescu A., Buraga I. (2016). The effect of levodopa-carbidopa intestinal gel infusion long-term therapy on motor complications in advanced Parkinson’s disease: A multicenter Romanian experience. J. Neural Transm..

[8] Timpka J., Fox T., Fox K., Honig H., Odin P., Martinez-Martin P., Antonini A., Ray Chaudhuri K. (2016). Improvement of dyskinesias with l-dopa infusion in advanced Parkinson’s disease. Acta Neurol. Scand..

[9] Antonini A., Fung V.S.C., Boyd J.T., Slevin J.T., Hall C., Chatamra K., Eaton S., Benesh J.A. (2016). Effect of levodopa-carbidopa intestinal gel on dyskinesia in advanced Parkinson’s disease patients. Mov. Disord..

[10] Lopiano L., Modugno N., Marano P., Sensi M., Meco G., Solla P., Gusmaroli G., Tamma F., Mancini F., Quatrale R. (2019). Motor and non-motor outcomes in patients with advanced Parkinson’s disease treated with levodopa/carbidopa intestinal gel: Final results of the GREENFIELD observational study. J. Neurol..

[11] Szász J.A., Constantin V.A., Orbán-Kis K., Rácz A., Bancu L.A., Georgescu D., Szederjesi J., Mihály I., Fárr A.-M., Kelemen K. (2019). Profile of Patients with Advanced Parkinson’s disease Suitable for Device-Aided Therapies: Restrospective Data of a Large Cohort Of Romanian Patients. Neuropsychiatr. Dis. Treat..

[12] Szász J.A., Szatmári S., Constantin V., Mihály I., Rácz A., Domokos L.C., Vajda T., Orbán-Kis K. (2019). Characteristics of levodopa treatment in advanced Parkinson’s disease in the experiences of the neurology clinics of Târgu Mures, Romania. Orv. Hetil..

[13] Szász J.A., Constantin V.A., Orbán-Kis K., Bancu L.A., Georgescu D., Szederjesi J., Rácz A., Mihály I., Szatmári S. (2018). Characteristics of dopaminergic treatments in advanced Parkinson’s before levodopa-carbidopa intestinal gel infusion: Data from 107 tested patients. Mov. Disord..

[14] Szasz J., Constantin V., Orban-Kis K., Bancu L., Georgescu D., Szederjesi J., Racz A., Mihaly I., Szederjesi J. (2019). Spectrum of motor complications in advanced Parkinson’s disease: Data from a large Romanian case series evaluated for suitability for device aided therapy. Mov. Disord..

[15] Tomlinson C.L., Stowe R., Patel S., Rick C., Gray R., Clarke C.E. (2010). Systematic review of levodopa dose equivalency reporting in Parkinson’s disease. Mov. Disord..

[16] Szasz J.A., Jianu D.C., Simu M.A., Constantin V.A., Dulamea A.O., Onuk K., Popescu D., Vasile M.T., Popescu B.O., Fasano A. (2021). Characterizing Advanced Parkinson’s Disease: Romanian Subanalysis from the OBSERVE-PD Study. Parkinson’s Dis..

[17] Constantin V.A., Szász J.A., Orbán-Kis K., Rosca E.C., Popovici M., Cornea A., Bancu L.A., Ciorba M., Mihály I., Nagy E. (2020). Levodopa-carbidopa intestinal gel infusion therapy discontinuation: A ten-year retrospective analysis of 204 treated patients. Neuropsychiatr. Dis. Treat..

[18] Krüger R., Hilker R., Winkler C., Lorrain M., Hahne M., Redecker C., Lingor P., Jost W.H. (2016). Advanced stages of PD: Interventional therapies and related patient-centered care. J. Neural Transm..

[19] Oertel W., Schulz J.B. (2016). Current and experimental treatments of Parkinson disease: A guide for neuroscientists. J. Neurochem..

[20] Luquin M.R., Kulisevsky J., Martinez-Martin P., Mir P., Tolosa E.S. (2017). Consensus on the Definition of Advanced Parkinson’s Disease: A Neurologists-Based Delphi Study (CEPA Study). Parkinson’s Dis..

[21] Antonini A., Stoessl A.J., Kleinman L.S., Skalicky A.M., Marshall T.S., Sail K.R., Onuk K., Odin P.L.A. (2018). Developing consensus among movement disorder specialists on clinical indicators for identification and management of advanced Parkinson’s disease: A multi-country Delphi-panel approach. Curr. Med. Res. Opin..

[22] Ahlskog J.E., Muenter M.D. (2001). Frequency of levodopa-related dyskinesias and motor fluctuations as estimated from the cumulative literature. Mov. Disord..

[23] Fabbri M., Ferreira J.J., Lees A., Stocchi F., Poewe W., Tolosa E., Rascol O. (2018). Opicapone for the treatment of Parkinson’s disease: A review of a new licensed medicine. Mov. Disord..

[24] Borgohain R., Szasz J., Stanzione P., Meshram C., Bhatt M., Chirilineau D., Stocchi F., Lucini V., Giuliani R., Forrest E. (2014). Randomized trial of safinamide add-on to levodopa in Parkinson’s disease with motor fluctuations. Mov. Disord..

[25] Borgohain R., Szasz J., Stanzione P., Meshram C., Bhatt M.H., Chirilineau D., Stocchi F., Lucini V., Giuliani R., Forrest E. (2014). Two-year, randomized, controlled study of safinamide as add-on to levodopa in mid to late Parkinson’s disease. Mov. Disord..

[26] Martí-Andrés G., Jiménez-Bolaños R., Arbelo-González J.M., Pagonabarraga J., Duran-Herrera C., Valenti-Azcarate R., Luquin M.R. (2019). Safinamide in clinical practice: A Spanish multicenter cohort study. Brain Sci..

[27] Szasz J.A., Szatmari S., Constantin V., Mihaly I., Racz A., Torok I., Nagy E., Kelemen K., Forro T., Baroti B. (2020). The importance of evaluation of gastrointestinal features in advanced Parkinson’s disease. Orv. Hetil..

[28] Patel A.B., Jimenez-Shahed J. (2018). Profile of inhaled levodopa and its potential in the treatment of Parkinson’s disease: Evidence to date. Neuropsychiatr. Dis. Treat..

[29] Szász J., Constantin V., Fazakas P., Blényesi E., Grieb L., Balla A., Sárig M., Szegedi K., Bartha E., Sz S. (2017). The role of selective monoamine oxidase B inhibitors in the therapeutic strategy of Parkinson’s disease in the neurology clinics of Tirgu Mures County Emergency Clinical Hospital. Orv. Hetil..

[30] Nyholm D., Odin P., Johansson A., Chatamra K., Locke C., Dutta S., Othman A.A. (2013). Pharmacokinetics of levodopa, carbidopa, and 3-O-methyldopa following 16-hour jejunal infusion of levodopa-carbidopa intestinal gel in advanced parkinson’s disease patients. AAPS J..

[31] Othman A.A., Dutta S. (2014). Population pharmacokinetics of levodopa in subjects with advanced Parkinson’s disease: Levodopa-carbidopa intestinal gel infusion vs. oral tablets. Br. J. Clin. Pharmacol..

[32] Szász J., Simu M., Perju-Dumbrava L., Antonini A., Bergmann L., Popescu D., Bajenaru O.A. (2020). Efficacy, safety and patient’s quality of life of long-term treatment with levodopa-carbidopa intestinal gel in advanced parkinson’s disease in romania: Results from gloria observational study. Rom. J. Neurol. Rev. Rom. Neurol..

[33] Müller T., Laar T. van, Cornblath D.R., Odin P., Klostermann F., Grandas F.J., Ebersbach G., Urban P.P., Valldeoriola F., Antonini A. (2013). Peripheral neuropathy in Parkinson’s disease: Levodopa exposure and implications for duodenal delivery. Park. Relat. Disord..

[34] Liu X.D., Bao Y., Liu G.J. (2019). Comparison between levodopa-carbidopa intestinal gel infusion and subthalamic nucleus deep-brain stimulation for advanced Parkinson’s disease: A systematic review and meta-analysis. Front. Neurol..

[35] Ricciardi L., Bove F., Espay K.J., Lena F., Modugno N., Poon Y.Y., Krikorian R., Espay A.J., Fasano A. (2016). 24-Hour infusion of levodopa/carbidopa intestinal gel for nocturnal akinesia in advanced Parkinson’s disease. Mov. Disord..

[36] Thakkar S., Fung V.S.C., Merola A., Rollins M., Soileau M.J., Kovács N. (2021). 24-Hour Levodopa-Carbidopa Intestinal Gel: Clinical Experience and Practical Recommendations. CNS Drugs.

[37] Cruse B., Morales-Briceño H., Chang F.C.F., Mahant N., Ha A.D., Kim S.D., Wolfe N., Kwan V., Tsui D.S., Griffith J.M. (2018). 24-hour levodopa-carbidopa intestinal gel may reduce troublesome dyskinesia in advanced Parkinson’s disease. NPJ Park. Dis..

[38] Morales-Briceño H., Mahant N., Ha A.D., Chang F.C.F., Kim S.D., Griffith J., Tsui D., Galea D., Fung V.S.C. (2019). Long-term safety and efficacy of 24-hour levodopa-carbidopa intestinal gel in Parkinson’s disease. Mov. Disord..

[39] Espay A.J., Morgante F., Merola A., Fasano A., Marsili L., Fox S.H., Bezard E., Picconi B., Calabresi P., Lang A.E. (2018). Levodopa-induced dyskinesia in Parkinson disease: Current and evolving concepts. Ann. Neurol..

[40] Guridi J., González-Redondo R., Obeso J.A. (2012). Clinical features, pathophysiology, and treatment of levodopa-induced dyskinesias in Parkinson’s disease. Parkinson’s Dis..

[41] Szász J.A., Szatmári S., Constantin V., Mihály I., Rácz A., Frigy A., Nagy E., Kelemen K., Forró T., Almásy E. (2021). Decision-making and duration to accept device-aided therapy in advanced Parkinson’s disease. Retrospective data from an Eastern European center with high patient turnover. Orv. Hetil..

[42] Poewe W., Chaudhuri K.R., Bergmann L., Antonini A. (2019). Levodopa-carbidopa intestinal gel in a subgroup of patients with dyskinesia at baseline from the GLORIA Registry. Neurodegener. Dis. Manag..

[43] Meloni M., Solla P., Mascia M.M., Marrosu F., Cannas A. (2017). Diphasic dyskinesias during levodopa-carbidopa intestinal gel (LCIG) infusion in Parkinson’s disease. Park. Relat. Disord..

[44] Marano M., Naranian T., di Biase L., Di Santo A., Poon Y.Y., Arca R., Cossu G., Marano P., Di Lazzaro V., Fasano A. (2019). Complex dyskinesias in Parkinson patients on levodopa/carbidopa intestinal gel. Park. Relat. Disord..

[45] Catalán M.J., Escribano P.M., Alonso-Frech F. (2017). Dyskinesias in levodopa-carbidopa intestinal gel infusion era: New challenges, new features. Mov. Disord..

[46] Fasano A., Gurevich T., Jech R., Kovács N., Svenningsson P., Szász J., Parra J.C., Bergmann L., Johnson A., Sanchez-Soliño O. (2021). Concomitant Medication Usage with Levodopa-Carbidopa Intestinal Gel: Results from the COSMOS Study. Mov. Disord..

[47] Zadikoff C., Poewe W., Boyd J.T., Bergmann L., Ijacu H., Kukreja P., Robieson W.Z., Benesh J., Antonini A. (2020). Safety of Levodopa-Carbidopa Intestinal Gel Treatment in Patients with Advanced Parkinson’s Disease Receiving ≥2000 mg Daily Dose of Levodopa. Parkinson’s Dis..

[48] Szász J.A., Orbán-Kis K., Constantin V.A., Péter C., Bíró I., Mihály I., Szegedi K., Balla A., Szatmári S. (2019). Therapeutic strategies in the early stages of Parkinson’s disease: A cross-sectional evaluation of 15 years’ experience with a large cohort of Romanian patients. Neuropsychiatr. Dis. Treat..

[49] Szász J.A., Constantin V., Mihály I., Biró I., Péter C., Orbán-Kis K., Szatmári S. (2019). Dopamine agonists in Parkinson’s disease therapy—15 years of experience of the neurological clinics from Tirgu Mures. A cross-sectional study. Ideggyogy. Szle..

