# Supplementary material for: Management Challenges of Severe, Complex Dyskinesia. Data from a Large Cohort of Patients Treated with Levodopa-Carbidopa Intestinal Gel for Advanced Parkinson’s Disease
Source: Brain Sci. 2021 Jun 22;11(7):826. doi: 10.3390/brainsci11070826 (PMC8301838; doi:10.3390/brainsci11070826)
Supplement: Supplementary file 1 [file brainsci-11-00826-s001.zip › brainsci-1255671-SI.pdf]

Video URL：https://zenodo.org/record/4812818#.YNUjg0wRVPY

Video 1 (RI)

A 55 years old patient, with 25 years disease duration, receiving levodopa/carbidopa for 25 years, 1500 mg/day divided in 6 doses, and pramipexole 2.1 mg/day.

The patient is presenting diphasic dyskinesias (also referred to as beginning- and end-of-dose or transitional dyskinesia), which have variable topographical distribution and are more disabling compared with peak-dose dyskinesias. They consist of repetitive, stereotypic movements of the extremities and the trunk and abrupt fluctuations between a good antiparkinsonian response and a severe parkinsonian motor state ("unpredictable on–off fluctuations", "sudden off").

Video 2 (VE)

61 y.o. patient with 14 years disease duration, receiving levodopa/carbidopa/entacapone for 14 years, 1200 mg/day divided in 10 doses and amantadine 200 mg/day.

The patient is presenting *off* period dystonia, characterized by fixed and painful postures more frequently affecting the feet, but which can be segmental or generalized in distribution

Video 3 (OM)

53 y.o. patient with 12 years disease duration, receiving levodopa/carbidopa/entacapone for 12 years, 500 mg/day divided in 5 doses, and ropinirole 8 mg/day, rasagiline 1 mg/day and amantadine 200 mg/day.

A combination of diphasic dyskinesia and off period dystonia was present in this patient throughout the entire LD cycle.
